# Inflammatory back pain associated with nail-patella syndrome: A case report

**DOI:** 10.1097/MD.0000000000043291

**Published:** 2025-07-04

**Authors:** Wen Chen, Tai Xu Lu, Yan Liu

**Affiliations:** a Department of Acupuncture and Rehabilitation, Dazu District Hospital of Traditional Chinese Medicine, Chongqing, China; b Department of Infection Management, Dazu District Hospital of Traditional Chinese Medicine, Chongqing, China.

**Keywords:** inflammatory back pain, nail-patella syndrome, radiology

## Abstract

**Rationale:**

Nail-patella syndrome (NPS), a rare genetic disorder with multisystemic implications that affects the kidneys, joints, bones, and nails, exhibits significant phenotypic heterogeneity, leading to diverse clinical presentations and prompting patients to seek medical attention. This report details the case of a male patient presenting with inflammatory back pain, whose subsequent diagnostic evaluations revealed skeletal anomalies consistent with NPS.

**Patient concerns:**

A male patient in his mid-thirties was diagnosed with inflammatory back pain associated with NPS.

**Diagnoses:**

The patient presented with hypoplastic nails, malformation of elbow joints, and dysplastic patellae. Computed tomography revealed an iliac angle deformity, which is a cardinal feature of NPS. The diagnostic criteria for NPS were based on clinical manifestations and computed tomography results.

**Interventions:**

The patient was prescribed celecoxib (0.2 grams orally once daily) for 1 week, along with physical therapy exercises.

**Outcomes:**

During the 3-month follow-up period, gradual improvement in the patient’s low back pain was observed.

**Lessons:**

This case underscores the importance of considering NPS in the differential diagnosis of inflammatory back pain.

## 1. Introduction

Nail-patella syndrome (NPS) is an autosomal dominant disorder with an approximate annual incidence of 1 in 50,000 individuals.^[1]^ Patients with NPS usually exhibit distinct clinical features such as nail dysplasia, patellar underdevelopment or absence, abnormal iliac angle formation, and radial head dislocation.^[2]^ Furthermore, associated symptoms such as glaucoma, cataracts, and proteinuria may also be present in some patients.^[3]^ In this paper, we report a case of the patient with NPS who complained of inflammatory back pain, aiming to enhance the understanding of this rare disorder’s diverse manifestations.

## 2. Case presentation

**Figure 1.**
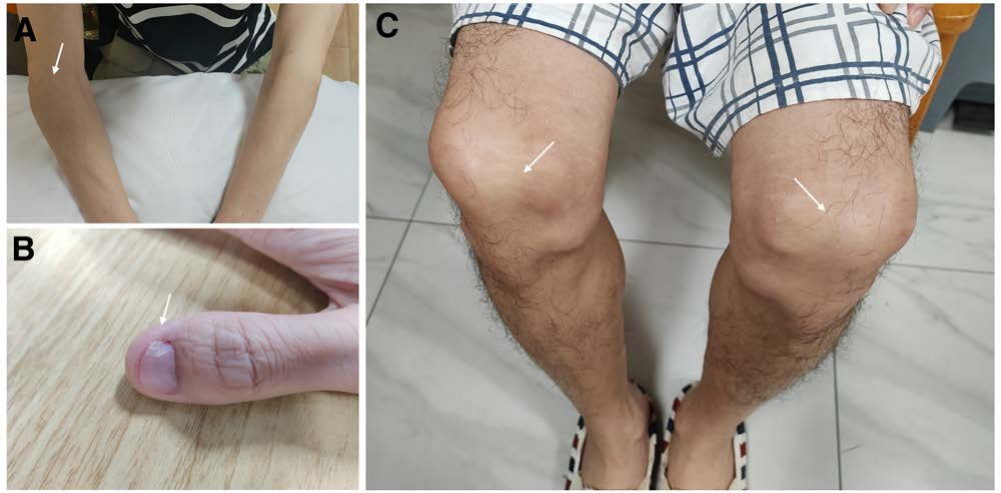
(A) The patient’s right elbow joint had limited extension. (B) Fissures could be seen on the patient’s fingernails. (C) The patient had abnormal patellae.

**Figure 2.**
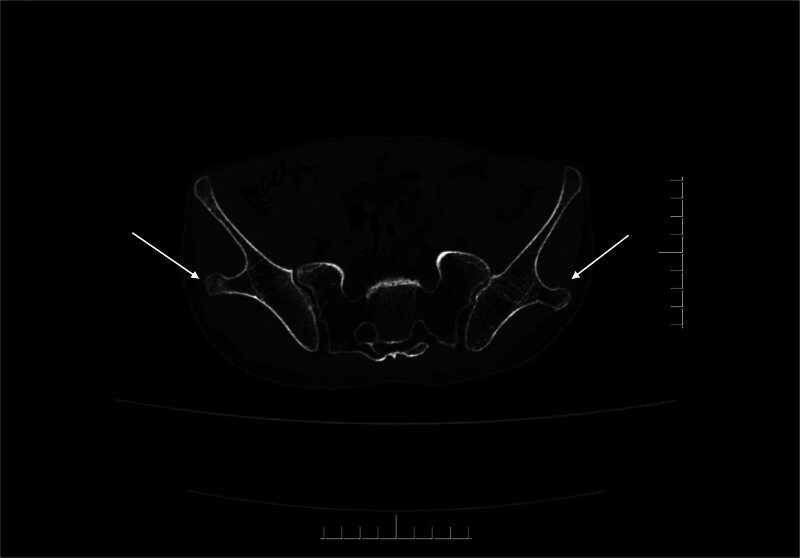
The “iliac horns” deformity, a characteristic manifestation of nail-patella syndrome, could be seen on the bilateral iliac bones of the patient.

**Figure 3.**
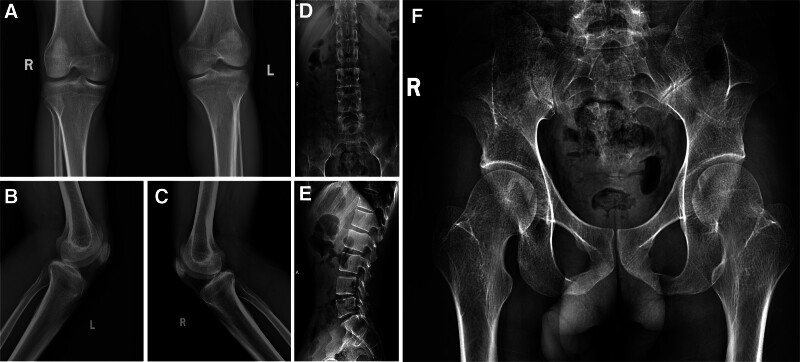
(A–C) AP and lateral knee radiographs. (D) AP lumbar spine radiograph. (E) Lateral lumbar. (F) AP pelvis radiograph. AP = anteroposterior.

A 30-year-old male suffered from low back pain for 8 years. He reported an increase in the frequency and severity of pain during the previous month, with pain peaking at night, which was relieved through physical exertion. Clinical examination revealed limited extension of his right elbow joint, along with fissures on his fingernails and patellar dysplasia (Fig. 1). Notably, his first-degree relatives exhibited similar nail and patella abnormalities. The Patrick test, used to assess hip flexion, abduction and external rotation, yielded normal outcomes. Simultaneously, the range of motion of the lumbar spine was unrestricted. Laboratory tests indicated that inflammatory markers and blood counts were within the normal range, and human leukocyte antigen B27 (HLA-B27) test was negative. Creatinine and urea levels were within the normal limits, and urinalysis was negative for proteins and cells (Table 1). Subsequently, a computed tomography (CT) was conducted to further explore the potential causes. Although the CT of sacroiliac joints showed no signs of sacroiliitis, iliac angles, a characteristic feature of NPS, were detected in the ilium region (Fig. 2). Subsequently, further imaging studies including anteroposterior and lateral views of both knees, lumbar spine, and plain pelvic radiographs were obtained (Fig. 3). These revealed underdeveloped patellae. After ruling out spondyloarthritis, the patient was ultimately diagnosed with NPS. Although genetic testing was recommended for both the patient and his family members, he declined due to financial constraints. He was prescribed celecoxib at a dosage of 0.2 g orally once daily for 1 week, accompanied by physical therapy exercises. During the 3-month follow-up period, his low back pain gradually improved. It is recommended that he should undergo annual screenings for blood pressure, urinalysis, intraocular pressure and glaucoma.

**Table 1:** The laboratory tests.

Variables	Results
Complete blood cell count	
White blood cell count (10^9^/L)	4.05
Lymphocyte (%)	33.6
Monocyte (%)	4.8
Eosinophil (%)	2.1
Red blood cell (10^9^/L)	5.35
Hemoglobin (g/L)	158
Platelet count (10^9^/L)	205
Liver and renal function	
Total bilirubin (μmol/L)	14.84
Total biliary acid (μmol/L)	3.6
Albumin (g/L)	50.2
Globulin (g/L)	26.4
ALT (U/L)	16
AST (U/L)	18
Creatinine (μmol/L)	70.8
Inflammatory markers	
High sensitivity C reaction protein (mg/L)	0.78
ESR (mm/h)	1
Urinalysis	
White blood cell	Negative
Red blood cell	Negative
Protein	Negative
Other	
HLA-B27	Negative

ALT = alanine aminotransferase, AST = aspartate aminotransferase, ESR = erythrocyte sedimentation rate, HLA-B27 = the human leukocyte antigen B27.

## 3. Discussion

Nail-patella syndrome, also known as hereditary nail-bone dysplasia, Turner-Kiser syndrome, or Fung syndrome, is a rare genetic disorder. The typical features of NPS encompass nail underdevelopment, patellar absence or dysplasia, iliac angular deformity, and elbow anomalies. It is commonly recognized that NPS is induced by heterozygous mutations in the LMX1B gene.^[4]^ However, in some patients, the coding sequence of LMX1B remains normal, suggesting other potential pathogenic mechanisms.^[5–7]^ The kidney is an organ often affected by NPS. Hematuria and proteinuria are prevalent among these patients, and a portion of them may progress to end-stage renal failure, which is the main cause of death related to NPS.^[8,9]^ Low back pain, another prevalent symptom of NPS, usually has its onset in childhood. Typically, the pain exacerbates after physical activity and is relieved by rest. This is associated with the augmented lumbar lordosis.^[3]^ In addition, glaucoma, cataracts, sensorineural deafness, and gastrointestinal discomfort are among the other possible symptoms. Although NPS is inherited in an autosomal dominant manner, there is significant variability in clinical manifestations among patients, even within the same family.^[10,11]^

Inflammatory back pain is more frequently seen in individuals with spondyloarthropathies, and it is characterized by the worsening of pain during nocturnal rest and the improvement in pain with physical activity. According to the criteria defined by the Assessment of SpondyloArthritis International Society (ASAS),^[12]^ the patient in this case had the features of inflammatory back pain. After completing the HLA-B27 test and sacroiliac joint CT scans, spondyloarthritis was effectively excluded, and a final diagnosis of NPS was made. This finding indicates that the consideration of rare diseases like NPS should be included in the differential diagnosis of patients with inflammatory back pain. Currently, there is no established curative treatment for NPS. However, angiotensin-converting enzyme inhibitors (ACEIs) have demonstrated efficacy in managing proteinuria.^[13]^ For osteoarticular symptoms, current treatment options include the use of analgesics,^[14]^ physical therapy, and surgical interventions.^[15]^

## 4. Conclusion

NPS is a rare genetic disorder that may manifest as inflammatory back pain. For patients who are unable to undergo genetic testing or have a negative result on LMX1B gene testing, imaging examinations are helpful in identifying the unique skeletal abnormalities associated with NPS.

## Acknowledgments

The authors thank all the staff for their valuable contribution to the study.

## Author contributions

**Conceptualization:** Wen Chen.

**Data curation:** Wen Chen, Tai Xu Lu.

**Formal analysis:** Wen Chen.

**Funding acquisition:** Wen Chen, Yan Liu.

**Investigation:** Wen Chen, Tai Xu Lu.

**Methodology:** Wen Chen, Yan Liu.

**Project administration:** Wen Chen, Yan Liu.

**Resources:** Wen Chen, Yan Liu.

**Writing – original draft:** Wen Chen, Tai Xu Lu.

**Writing – review & editing:** Wen Chen, Yan Liu.
